# Chemical composition of essential oil and anti trichomonas activity of leaf, stem, and flower of *Rheum ribes* L. extracts

**Published:** 2014

**Authors:** Forough Naemi, Gholamreza Asghari, Hossein Yousofi, Hossein Ali Yousefi

**Affiliations:** 1 ^*‎*^*Department of Pharmacognosy, Faculty of Pharmacy, Isfahan University of Medical Sciences, Isfahan, I. R. Iran*; 2 *Isfahan Pharmaceutical Sciences Research Center, Isfahan University of Medical Sciences, Isfahan, I. R. Iran*; 3 *Department of Parasitology, Faculty of Medicine, Isfahan University of Medical Sciences, Isfahan, I. R. Iran*

**Keywords:** *Essential oil*, *Rheum ribes*, *Trichomonas vaginalis*

## Abstract

**Objectives**: Trichomoniasis is one of the most common sexually transmitted diseases in humans and is caused by the protozoan Trichomonas vaginalis. Nowadays, increasing resistance to drugs such as metronidazole resulted in many problem, so new effective remedies are needed. In this study, we evaluate constituents of essential oil and anti-trichomonas activity of Rheum ribes.

**Materials and Methods**: The essential oil from Rheum ribes L. flower growing wild in Iran was analyzed by GC/MS. The parasites were treated with different extract and fractions of the flower, stem, and leave of the plant. Anti-trichomonas activity was evaluated using an in vitro assay.

**Results**: In all, 19 compounds were identified; palmitic acid [27.08%], n-eicosane [9.9%], n-tetracosane [7.34%], linoleic acid [6.56%], and ethyl linoleate [4.76%] were the main components of the oil. Rheum ribes extracts and fractions concentration dependently inhibited the ability of parasites to growth. This was associated with polarity of solvent used for fractionation and plant parts used for extraction.

**Conclusion**: Findings demonstrate the potential of Rheum ribes extracts as an anti-trichomonas agent for human use. Further studies are required to evaluate its toxicity and safety.

## Introduction

 Volvovaginitis as a common medical problem can lead to considerable discomfort and repeated medical visits. Infection, allergy, and systemic diseases can happen due to volvovaginitis. Its main causes before menopause are bacterial vaginitis, volvovaginal candidiasis, and *Trichomonas vaginalis* (Nyirjesy et al., 2006 ▶). Trichomoniasis infects more than 100 million new people every year and it is the most prevalent non-viral sexually infection. Infant mortality, preterm delivery or low birth weight, and susceptibility to HIV infection are related to trichomoniasis(Schwebke and Burgess, 2004 ▶; Upcroft and Upcroft, 2001 ▶; Patel et al., 2000 ▶; Petrin et al., 1998 ▶).

Metronidazole is the approved drug for the treatment of trichomoniasis, but some resistant strains have been detected (Edwards, 1993 ▶; Grossman and Galask, 1990 ▶). In addition, it is inhibited in the first trimester of pregnancy, drug allergy, and sometimes for side effects. Therefore, we need to find new alternative drugs for trichomoniasis control. In all over the world, there is a widespread acceptance for using medicinal herbs. Herbal remedies have significant benefits such as fewer side effects, better patient tolerance, and better accessibility (Vermani and Garg, 2002 ▶).

*Rheum ribes* L. is a member of the polygonaceae family which is distributed in Iran and a few neighboring countries (Fazli-bazaz et al., 2005 ▶). It is a perennial and cultivated in some temperate countries for its edible red leaf stalks (Dehkardy, 2002 ▶; Zargari, 1991 ▶). Its Persian name is “Rivas” (Zargari, 1991 ▶). Traditionally, it has been used in Iran as sedative and mood enhancer (Sayyah et al., 2009 ▶) and in Turkey for the treatment of urinary inflammation and as a diuretic agent (Cakilcioglu and Turkoglu, 2010 ▶). In Iran, the plant roots are used as oriental laxative medicine and an anti-psoriatic drug (Shokravi and Agha Nasiri, 1997 ▶). Fresh stems and petioles are used as appetizer and digestive in Turkey. Its rhizomes are used to treat hypertension, obesity, and kidney sand and stones (Abu-Irmaileh and Afifi, 2003 ▶). Young shoots and petioles are consumed against diarrhea, stomachic, and antiemetic. It is used against hemorrhoids, measles, smallpox, and cholagogue (Baytop, 1999 ▶) and also against ulcer, as anti-helmintic, and expectorant (Tabata et al., 1994 ▶). The stem and root have been used for the treatment of anemia, anorexia, weakness, anxiety, and it has good effects on major depressive disorder (Sayyah et al., 2009 ▶). Its hydroalcoholic extract has some effective in treatment of obsessive compulsive disorders (Sayyah et al., 2011 ▶). 

It has a significant hypoglycemic effect due to anthraquinone glycosides of aloe emodin, emodin, physcion, and chrysophanol derivatives (Naqishbandi et al., 2009 ▶; Abu-Irmaileh and Afifi, 2003 ▶; Tabata et al., 1994 ▶) and it can be considered as a potential candidate for melioration/management of type II diabetes (Kasabri et al., 2011 ▶). It can decrease plasma lipids at the rank of or more than nicotinic acid (Hadjzadeh et al., 2004 ▶; Hajzadeh and Jafari, 2004 ▶). 

It was reported that, it has good effects on lipid as well as glucose profile in type II diabetic hypercholesterolemia patients without any adverse effect on kidney and liver (Falah Hosseini et al., 2008 ▶). Moreover, it was demonstrated to have antimicrobial activity against *Bacillus subtilis* and *Enterobactera erogenes* (Alan et al., 2012 ▶) *Staphylococcus aureus *(Alaadin et al., 2007 ▶) *P. aeruginosa*, and *Proteus spp* (Fazli-bazaz et al., 2005 ▶). Moreover, it is showed to possess anti-HSV activity (Hudson et al., 2000 ▶). Flower extract of the plant effectively reduced disease incidence of cumin wilt (Ghorbany and Salary, 2005 ▶). Some studies showed an antioxidant effect of *R. ribes* (Ozturk et al., 2007 ▶) and the anticholinesterase effect (50%) due to both terpenoides and alkaloids (Gholamhoseinian et al., 2012 ▶; Gholamhoseinian et al., 2009 ▶). The *R. ribes* is considered a rich source of vitamins A, B, C, and E (Falah Hosseini et al., 2008 ▶) and also minerals such as aluminum, calcium, iron, potassium, magnesium, sodium, phosphorus, zinc (Ozcan et al., 2007 ▶), cupper (Andiç et al., 2009 ▶), and selenium (Munzuroglu et al., 2000 ▶). Chemical studies on the roots of *R. ribes* showed presence of chrysophanol, physcion, rhein, aloeemodin, physcion-8-O-glucoside, aloeemodin-8-O-glucoside, sennoside A, rhaponticin (Tuzlaci and Meriçli, 1992 ▶; Meriçli and Tuzlaci, 1990 ▶), and flavonoids (Octay et al., 2007 ▶; Zargari, 1991 ▶). 

In this study, we examined *in vitro* activity of plant part extracts to figure out which part is more efficiently active against *Trichomonas vaginalis.*

## Materials and Methods

**Plant materials**

The fresh leaves, stems and flowers of Rheum ribes in flowering stage were collected in May 2012 from the Alvand mountain, altitude 3111 m, Isfahan province, Iran. The plant was recognized by Mr. Parvazian, technical officer of Faculty of Pharmacy, Isfahan University of Medical Sciences, Iran. The voucher specimen was deposited in the herbarium of faculty of pharmacy and Pharmaceutical Sciences (Voucher specimen No. 2291). Then, it was cut to pieces, air dried for seven days, and were ground into powder. 

**GC –MS Analysis**

The air-dried flower of the plant was powdered. The essential oil of powdered flower was obtained using hydro-distillation. 

Then essential oil was injected to GC-Mass apparatus (Agilent 6890) equipped with a HP-5MS fused silica column (30×0.25 mm^2^: film thickness 0.25 µm) and interfaced with a Agilent 5975 mass selective detector. The oven temperature was programmed from 60 -280 °C at rate of 4 °C/min. Helium was used as carrier gas at a flow rate of 2 ml/min. Other conditions of the instrument were as follows: ionization voltage 70 eV, injector temperature 280 °C, and ion source temperature 200 °C. Identification of components of oil were based on GC retention indices relative to n-alkenes and computer matching with the WILEY275.L library, as well as by comparison of the fragmentation patterns of the mass spectra with those reported in the literature (Adams, 1995 ▶; Davies, 1990 ▶).

**Extraction and fractionation**

The crude extract was prepared bymaceration of plant leaves, stems, and flowers powder (200 gr) with methanol (1000 ml) in an air tight container for 7 days at room temperature with occasional shaking. Then, the extracts were filtered through a cotton plug following with a Whatman filter paper. Using a rotatory evaporator, the extracts were concentrated at low temperature and pressure. The crude methanol extracts (10 ml) were reserved for evaluation of anti-trichomonas activity. The remaining extract were mixed with methanol (90 ml) and water (10 ml) and then partitioned using n-hexane (3×100 ml) and dichloromethane (3×100 ml), sequentially. Finally, 11 fractions were obtained including total extract (methanol), hexane fraction, dichloromethane fraction, and residual aqueous fraction. The extracts and fractions were dried in room temperature. The dry extracts were kept in air tight containers for *in vitro* anti-trichomonas screening (Muhit et al., 2010 ▶).

**Preparation of test microorganism**

*Trichomonas vaginalis* was obtained from parasitology lab of Isfahan University of Medical Science, which was isolated from vaginal discharge of female patients attending to Obstetric and Gynecology Clinic at Shahrekord. This parasite was cultured *in vitro* at 37 °C in TYIS33. Log phase culture was diluted with TYIS33 medium for obtaining 10000 cell/ml. Extracts and fractions were diluted with water to obtain final concentration of 0.125, 0.25, 5, and 10 mg/ml and then the extracts and fractions (100 µl) were mixed with parasite (10 µl) + medium (890 µl). Metronidazole (100 µl) + parasite (10 µl) + medium (890 µl) were used as positive control. Medium (100 µl + 890 µl = 990µl) + parasite (10 µl) was used as negative control. 

All tubes were incubated at 37 °C. After 24 and 48 hours, samples were taken from each tube and viable parasites, completely active and flagella active were counted with hemocytometer in triplicate. Results of counting are reported as percentage of growth inhibition (G.I %) using following equation:

$\mathrm{G.I \%}:=\frac{a-b}{a}\times 100$

a: mean number of viable parasites in negative control tube.

b: mean number of viable parasites in test tube (Tonkal, 2009 ▶; Moon et al., 2006 ▶).

## Results

Table 1 lists retention indices, Kovat indices (KI), and percentage of constituents in the essential oil of *Rheum ribes.* GC-MS analysis of *R. ribes* essential oil resulted in the detection of 19 components consisting of 8 alkanes, 5 monoterpenes, 3 fatty acid esters, 2 fatty acids, and 1 sesquiterpene.

**Table 1:** The composition of *Rheum ribes* L. flower essential oil

**Peak No.**	**Component**	**Classification**	**Retention Index**	**% in oil**	**Kovats ** **Index**
1	Myrcene	Hydrocarbon monoterpene	3.79	Trace	0991
2	[+]-3-carene	Hydrocarbon monoterpene	5.32	Trace	1011
3	1,8 Cineole	Alcohol monoterpene	5.84	0.87	1033
4	Camphor	Ketone monoterpene	8.90	Trace	1143
5	Bornyl acetate	Ester monoterpene	13.23	Trace	1285
6	Aromadendrene	Hydrocarbon sesquiterpene	22.40	Trace	1439
7	n-Hexadecane	Alkane	30.56	Trace	1600
8	Palmitic acid	Saturated fatty acid	32.31	27.08	1971
9	Ethyl hexadecanoate	Ester of fatty acid	32.86	1.30	1993
10	n-Eicosane	Alkane	35.26	9.90	2000
11	Linoleic acid	Unsaturated fatty acid	36.06	6.56	2136
12	Ethyl linoleate	Ester of fatty acid	36.63	4.76	2162
13	9,12,15-Octadecatrienoic acid methyl ester	Ester of fatty acid	36.77	4.01	2168
14	n-Tricosane	Alkane	37.45	1.57	2300
15	n-Tetracosane	Alkane	39.61	17.81	2400
16	n-Octacosane	Alkane	41.59	1.33	2800
17	n-Nonacosane	Alkane	43.53	7.34	2900
18	n-Triacontane	Alkane	47.24	3.90	3000
19	Tetratetracontane	Alkane	50.65	2.15	4400

Anti-trichomonas activity revealed that, no growth was observed after 24 and 48 h of incubation at 1 mg/ml concentration of all extracts and fractions. Subsequently, it was observed that almost all cells were dead at 24 h of incubation at this concentration. However, at lower concentrations of 0.125 mg/ml, the cells were viable even after 48 h of incubation. Viability and number of trichomonas were reduced by several concentrations of extracts and fractions with no live parasites at 0.25 mg/ml for water fraction of flower (Table 2).

With no exception, all the fractions showed greater activity at 48 h than at 24 h probably due to increased contact time of the compounds with the organisms. Although, some fractions were less active than the standard drug metronidazole. 100% of growth inhibition in positive control tube containing metronidazole at first 24 h were observed. The negative control tube showed 0% growth inhibition.

**Figure 1:**
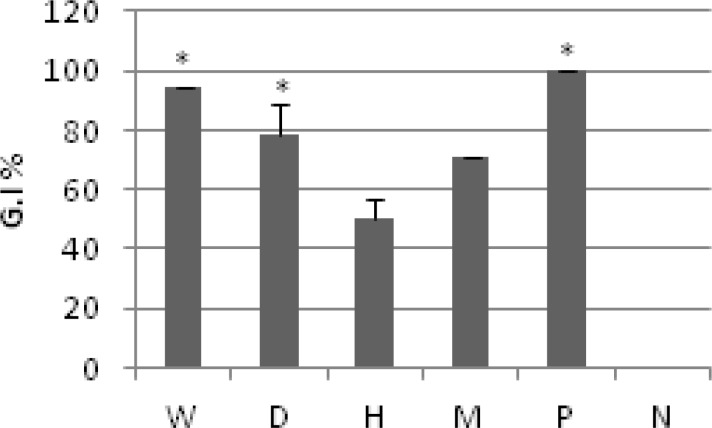
Comparison of anti-trichomonas activity of several fractions (0.25 mg/ml) of *R. ribes *flower at 24 h*.* Values are presented as mean±SD. *p* value<0.05. Water (W), Dichloromethane (D), Hexane (H), Methanol (M), Positive control (P), and Negative control (N).

Comparison of anti-trichomonas activity of several fractions (0.25 mg/ml) of *R. ribes *flower at 24 h is presented in Figure 1.

**Figure 2:**
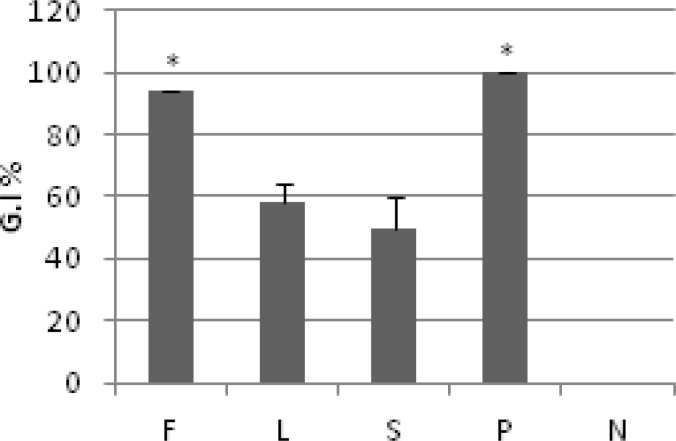
Comparison of anti-trichomonas activity of different plant parts (0.25 mg/ml) of *R. ribes *at 24 h*.* Values are presented as mean±SD. *p* value<0.05. Flower (F), Leaf (L), Stem (S), Positive control (P), and Negative control (N).

**Table 2:** Anti-trichomonas effect of different extracts and fractions obtained from several parts of *R. ribes*

**Growth Inhibition (%) at different time intervals**		**Concentration (mg/ml)**	**Fraction**	**Plant part**
**48h**	**24 h**			
90.8 ± 1.70	74.28 ± 4.04	0.125	Water	**Flower**
99.7 ± 0.14	94.28 ± 0	0.25		
100 ± 0	100 ± 0	0.5		
100 ± 0	100 ± 0	1		
67.86 ± 0.25	57.14 ± 0	0.125	Dichloromethane	
88.39 ± 1.26	78.56 ± 10.10	0.25		
100 ± 0	92.86 ±10.10	0.5		
100 ± 0	100 ± 0	1		
81.30 ± 1.62	66.66 ± 0	0.125	Methanol	
96.18 ± 0	71.42 ± 0	0.25		
98.47 ± 0	78.57 ± 3.36	0.5		
100 ± 0	100 ± 0	1		
88.10 ± 2.78	13.79 ± 0	0.125	Hexane	
96.76 ± 0.33	50 ± 7.32	0.25		
99.76 ± 0.11	84.48 ±2.44	0.5		
100 ± 0	100 ± 0	1		
83.38 ± 0.53	65.71 ± 4.04	0.125	Water	**Leaf**
86.12 ± 0.88	58.56 ± 6.06	0.25		
91 ± 0.71	94.28 ± 0	0.5		
100 ± 0	100 ± 0	1		
78.46 ± 0.93	23.07 ± 0	0.125	Dichloromethane	
98.57 ± 0.16	57.68 ± 5.44	0.25		
99.45 ± 0.16	94.22 ± 2.72	0.5		
100 ± 0	100 ± 0	1		
91.53 ± 0.78	40 ± 0	0.125	Methanol	
95.71 ± 0.47	52 ± 5.66	0.25		
100 ± 0	100 ± 0	0.5		
100 ± 0	100 ± 0	1		
87.98 ± 2.04	65 ± 7.07	0.125	Hexane	
95.67 ± 2.04	45 ± 35.36	0.25		
99.52 ± 0.68	80 ± 0	0.5		
100 ± 0	100 ± 0	1		
16.22 ± 6.08	42.85 ± 0	0.125	Water	**Stem**
32.78 ± 10.77	50 ± 10.10	0.25		
70.52 ± 5.15	71.42 ± 0	0.5		
100 ± 0	100 ± 0	1		
16.48 ± 6.22	68 ± 0	0.125	Methanol	
90.22 ± 0.46	82 ± 2.83	0.25		
96.92 ± 0	82 ± 8.48	0.5		
100 ± 0	100 ± 0	1		
64.56 ± 3.27	21.42 ± 10.10	0.125	Hexane	
87.42 ± 1.87	57.14 ± 0	0.25		
100 ± 0	100 ± 0	0.5		
100 ± 0	100 ± 0	1		
100 ± 0	100 ± 0	0.1	Metronidazol	**Positive control**

**Statistical analysis**

Results are expressed as mean plus/minus standard deviation and the minimal level of significance was considered at p<0.05. All statistical analyses were assessed using SPSS statistical version 17.0 software. Differences among groups were tested by parametric one-way analysis of variance (ANOVA) with Tukey’s post-hoc test. 

## Discussion

As presented in Table 1, the major constituents of *R. ribes *flower essential oil were palmitic acid (27.08%), n-eicosane (9.9%), n-tetracosane (7.34%), linoleic acid (6.56%), and ethyllinoleate (4.76%). The interesting point to note was that the main component of *Rheum palmatum* root essential oil was palmitic acid (20.25%) (Miyazawa et al., 1996 ▶). It seems that fatty acids are the superior components of *Rheum* genus essential oil.

As presented on Table 2, there are fluctuations on anti-trichomonas activity according to plant part extract and solvent used for extract fractionation. Metronidazole has been known as the most effective drug for treatment of *Trichomonas vaginalis*-related diseases. However, it has been reported that metronidazole has adverse effects and incidence of metronidazole-resistant *T. vaginalis *has increased. Development of new drugs, which are effective against metronidazole-resistant *T. vaginalis* has been required. 

As presented in Table 2, water fraction of flower of *R. ribes *is shown to possess the highest percentage of growth inhibition (G.I=100%) with the least concentration (0.5 mg/ml) after 24 h in comparison with positive control. Similarly, 100% of growth inhibition was reported after 24 h of *T. vaginalis* exposure to *Nigella sativa* aqueous extract in concentration of 10 mg/ml (Tonkal, 2009 ▶). It was reported that 500 µg/ml ethyl acetate extract of *Arbutus anedo* leaves showed 100% growth inhibition (Ertabaklar et al., 2009 ▶). Moreover, the polar compounds including phenolic materials were more effective than semi-polar and non-polar compounds in *Eucalyptus camaldulensis *(Hassani et al., 2013 ▶). It was also shown that other polar compounds such as allicin and ajoene which exist in *Allium hirtifolium* could exhibit anti-trichomonas activity in comparison with metronidazole (Taran et al., 2006 ▶). In addition, researchers reported that berberine isolated from *Berberis asisata* had *in vitro *activity compared with metronidazole on *T. vaginalis* (Soffar et al., 2001 ▶). Moreover, alcoholic extracts of *Calendula officinalis* and *Echinacea angustifolia* had *in vitro* efficacy against *T. vaginalis* (Samochowiec et al., 1979 ▶). 

Various herbal medications have been used to kill *T. vaginalis in vitro*. Eighteen aqueous and two ethanol extracts of traditional herbal medicines used to treat trichomoniasis in the Republic of Korea were assessed for their anti-trichomonas activities. Two extracts (*Sophorae radix*, *Phellodendri cortex*) showed evident anti-trichomonas activity at 8 mg/ml. As presented on Figure 1, anti-trichomonas activity of several fractions of *R. ribes *flower at concentration of 0.25 mg/ml was varied. The ethyl acetate fraction of *Sophorae radix* showed anti-trichomonas activity at only 400 µg/ml (Park et al., 2005 ▶). 

The water fraction of *R. ribes *flower showed anti-trichomonas activity at 0.125 mg/ml. These findings indicate that *R. ribes *flower is a potent anti-trichomonas agent. In addition, Kim et al. (2003) ▶, Choi et al. (2002) ▶, and Park et al. (2004) ▶ found that a kalopanax saponin A isolate from *Kalopanax pictus* and extracts of *Sophora flavescens* and *Gleditsia sinensis* have anti-protozoal effects on *T. vaginalis* by inhibiting cell growth and impairing protein synthesis, respectively. In the aerial parts of *R. ribes*, chrysophanol, physcion, emodin, quercetin, 5-desoxyquercetin, quercetin 3-O-rhamnoside, quercetin 3-O-galactoside, and quercetin 3-O-rutinoside have been found (Tosun and Akyu¨z-Kızılay, 2003 ▶). Ryang et al. (2001) ▶ found that extract of *Gentiana scabra* var *buergeri* can also inhibit the growth of *T. vaginalis*. In general, anti trichomonas activity of plant materials seems to depend on solvent and plant used for extraction. Difference in growth inhibition concentration in these plants could be due to different chemical composition.

In conclusion, the present study demonstrated that extracts and fractions obtained from the extracts of different parts of *R. ribes *exhibit different anti-trichomonas activity at several concentrations and can suggest potential use of *R. ribes* flower water extract for development of an anti-trichomonas drug for human use. Further studies are, however, are required to evaluate its toxicity and safety.
